# IgE-Dependent Allergy in Patients with Celiac Disease: A Systematic Review

**DOI:** 10.3390/nu15040995

**Published:** 2023-02-16

**Authors:** Emilia Majsiak, Magdalena Choina, Izabela Knyziak-Mędrzycka, Joanna Beata Bierła, Kamil Janeczek, Julia Wykrota, Bożena Cukrowska

**Affiliations:** 1 Department of Health Promotion, Faculty Health of Sciences, Medical University of Lublin, Staszica 4/6, 20-081 Lublin, Poland; 2 Polish-Ukrainian Foundation of Medicine Development, Nałęczowska 14, 20-701 Lublin, Poland; 3 Outpatient Allergology Clinic, The Children’s Memorial Health Institute, Aleja Dzieci Polskich 20, 04-730 Warsaw, Poland; 4 Department of Pathomorphology, The Children’s Memorial Health Institute, Aleja Dzieci Polskich 20, 04-730 Warsaw, Poland; 5 Department of Pulmonary Diseases and Children Rheumatology, Medical University of Lublin, Profesora Antoniego Gębali Street, 20-093 Lublin, Poland; 6 Department of Biostatistics and Translational Medicine, Medical University of Lodz, Mazowiecka 15, 92-215 Lodz, Poland

**Keywords:** celiac disease, IgE-mediated allergy, immunoglobulin E

## Abstract

In order to answer the question if an IgE-mediated allergy (A-IgE) may occur in subjects with celiac disease (CD), a systematic review was performed of available publications collected in the United States National Institute for Biotechnology Information/National Institutes of Health/National Library of Medicine/PubMed database up to 28 December 2022, with the use of the following keywords “allergy&celiac/coeliac”, “sensitization&celiac/coeliac”, and “anaphylaxis&celiac/coeliac” compared in the form of a conjunction. In total, the search returned 2013 publications from these keywords in any section of the article. As numerous review articles included the above-mentioned entries in the abstract, we decided to focus on the publications with the entries only in the title (n = 63). After rejecting studies unrelated to the topic, narrative reviews, book chapters, conference abstracts, symposium reports, letters to the editor, or non-English articles, 18 publications (6 observational original studies and 12 case reports describing a total of 15 cases of A-IgE developed after a diagnosis of CD) were included to this review. Our study is the first systematic review on allergy occurrence in CD patients. The analysis indicated that the possibility of a coexistence of A-IgE with any food and inhalant allergens in subjects diagnosed with CD should be considered. A sensitization to wheat was the most frequently described in subjects with CD. The clinical manifestation of A-IgE in CD was similar to that in subjects without CD; e.g., with possible atopic dermatitis, vomiting, urticaria, angioedema, or anaphylactic shock. Screening for allergies in subjects with CD should be considered, especially in those cases where symptoms persist after introducing a gluten-free diet. The elimination of wheat from the diet of patients with CD may lead to a loss of immune tolerance and to the development of sensitization, which may even manifest as anaphylaxis. In conclusion, although there are few studies assessing the occurrence of A-IgE in subjects with CD, they show the possibility of a coexistence of both diseases and the high clinical significance of this phenomenon, which indicates the need for further studies.

## 1. Introduction

Celiac disease (CD) is one of the most common autoimmune diseases and is characterized by non-specific symptoms both from and beyond the gastrointestinal tract [1]. Gastrological symptoms mainly include stomach aches, bloating, diarrhea, or constipation. Symptoms beyond the gastrointestinal tract may include chronic fatigue, headaches, and anemia [2,3]. The basic and most effective method of CD treatment is a gluten-free diet (GFD) [4]. However, it has been shown that in more than 80% of subjects, the symptoms may persist despite a restrictive GFD [5]. The studies of our team have revealed that symptoms in Polish CD patients persisted for up to 6 years after introducing a GFD [5,6]. One of the possible causes of persisting symptoms despite the use of a GFD may be the coexistence of other diseases. In the above-mentioned study, the respondents most frequently mentioned anemia (34%) and allergies (13%) when asked about comorbidities.

Although anemia is quite common in CD, posing a problem if it is a symptom or a complication of the disease [7], the literature data on the coexistence of CD and allergies are rather scarce. 

An allergy is described as an exaggerated response from the body’s immune system to otherwise inert substances present in the environment. An allergy may be IgE-mediated, non-IgE-mediated, or mixed (IgE-dependent and non-IgE-dependent at the same time). Possible symptoms that may occur are itching; redness; a rash; hives; swelling of the lips, tongue, and airways (angioedema); nausea; vomiting; abdominal cramps; shortness of breath; wheezing; bronchospasms; stridor; fainting; or collapse [8]. Anaphylaxis is the most severe form of a hypersensitivity reaction, the very rapid progression of which can be life-threatening and fatal without an intervention [9].

Both CD and allergies are related to an abnormal function of the immune system, especially of the regulatory T cells in the gastrointestinal tract, which play a fundamental role in maintaining immune tolerance and preventing both autoimmunization and sensitization [10].

The proper development of the immune tolerance of a given organism determines that the food it consumes is not treated as a potentially pathogenic antigen [8]. However, in a few situations, the disturbance of this tolerance and the stimulation of the immune system with a food antigen may lead to an abnormal immune reaction and result in the occurrence of, for example, CD and/or a food allergy (FA) [8,11]. The clinical symptoms and biochemical abnormalities ameliorate after an elimination diet in both conditions [4]. A lifelong gluten-free diet and a diet with the temporary elimination of harmful food allergens are prescribed for CD and FA, respectively [12].

The coexistence of CD and allergies is not fully explained and the literature lacks data on the frequency of the coexistence of these diseases as well as the population distribution or clinical significance. That is why, in the present study, we aimed to review the available publications for the occurrence of an IgE-mediated allergy (A-IgE) in subjects with CD.

## 2. Materials and Methods

This systematic review was performed in accordance with Preferred Reporting Items for Systematic Reviews and Meta-Analysis (PRISMA) guidelines [13]. The selected papers were important to reach the research question: “Can A-IgE occur in CD patients?”. The electronic search was performed by two pairs of independent reviewers. Any disagreement was resolved by a discussion until a consensus was reached or by consulting a third author. After the search, all articles were inserted into the EndNote program to exclude duplicate articles and then saved in an Excel data sheet, where the classification, selection, and screening took place depending on the type of study, year, and relevance. Subsequently, an evaluation of the titles and abstracts was performed that screened the papers according to the eligibility criteria for the full-text evaluation and then determined the included studies for the qualitative evaluation. The descriptive data of the clinical or methodological factors such as the location, type of study, sample, age, type of allergen, symptoms, type of tests used to diagnose allergies, and prevalence results were extracted. In the case of lost or confusing data, the authors were contacted via e-mail. In cases of discussions, the authors were contacted by e-mail. The review protocol was submitted for registration to the PROSPERO system with the number CRD42023384887.

Due to the limited number of original studies included in our analysis, the low precision of the effect sizes, and high variability of the data, calculations of the bias with the use of statistical methods based on asymmetry and regression such as funnel plots or Egger’s test were not performed.

### 2.1. Search Strategy

In order to verify the possible occurrence of A-IgE with any food and inhalant allergens in patients with CD, available publications on this issue gathered in the United States National Institute for Biotechnology Information/National Institutes of Health/National Library of Medicine/PubMed database (https://www.ncbi.nlm.nih.gov/pubmed) accessed on 28 December 2022 were collected and reviewed. The search was based on the following keywords: “allergy&celiac/coeliac”, “sensitization&celiac/coeliac”, and “anaphylaxis&celiac/coeliac” compared in the form of a conjunction. Due to the multiplicity of the review articles on gluten-related diseases where the keywords often occurred in the abstract, it was decided to search for the above-mentioned entries only in the titles of the articles. 

### 2.2. Inclusion and Exclusion Criteria

**Figure 1:**
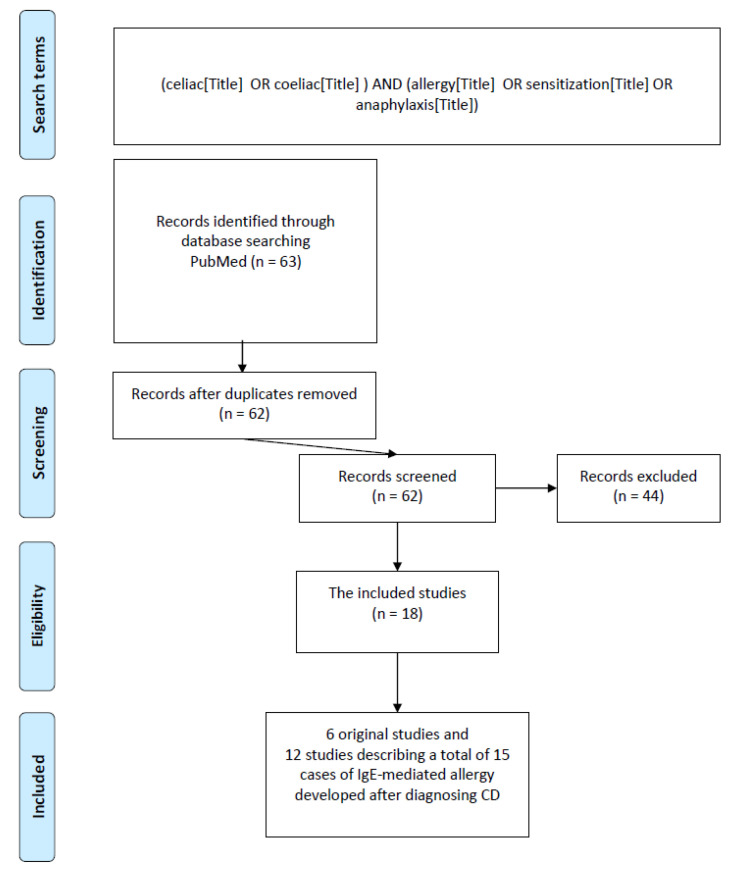
Flow chart showing the process for inclusion of studies [13].

The inclusion criteria were as follows, based on peer-reviewed full-text papers: systematic reviews and meta-analyses; original articles; case studies; written in English; and a description of the presence of an allergy, sensitization, or anaphylaxis in children and/or adults with CD that was related to the research question (Figure 1). The exclusion criteria were papers not related to the topic, narrative reviews, papers not available in full-length, written in languages other than English, animal studies, conference abstracts, editorials, letters to the editor, book chapters, symposium reports, or articles describing only the psychological issues related to A-IgE and CD.

## 3. Results

In total, a search using the search keywords (“allergy&celiac/coeliac”, “sensitization&celiac/coeliac”, and “anaphylaxis&celiac/coeliac”) returned 2013 publications. After narrowing down the search to the content of these keywords in the titles, we obtained 63 publications (53 for “allergy&celiac/coeliac” keywords, 7 for “sensitization&celiac/coeliac”, and 3 for “anaphylaxis&celiac/coeliac”). From a total of 53 articles containing the words “allergy&celiac/coeliac”, 5 of them were deleted (4 articles, although containing the searched words, did not refer to the research topic after reading the content, and 1 was dated 2023). From the whole number of found articles, we also deleted one article with the use of “sensitization&celiac” and with “allergy&celiac” because its title contained all three words “allergy&sensitization&celiac”. After rejecting the articles that were not related to the topic and did not meet the inclusion criteria, 18 publications remained: 6 original papers (observational studies) and 12 studies describing a total of 15 cases of A-IgE developed after a diagnosis of CD. These were included in the analysis. No systematic reviews or meta-analyses were found in the obtained materials. The number of obtained publications is presented in Table 1.

### 3.1. Incidence of A-IgE/Sensitization in Patients with CD 

An analysis of six original studies (Table 2) revealed a possible coexistence of CD and A-IgE in four of them; however, two of them did not confirm this correlation. Among the papers confirming the possibility of an A-IgE occurrence in subjects with CD, two papers concerned allergies [14,15] and another two concerned sensitization [12,16]. Among the studies included in the analysis, there were four prospective [14,15] and two retrospective research studies [12,16]. Five of the six studies were conducted in Europe [12,14,15,17,18]. The study groups varied significantly and included 57 to 1044 people with CD [14,15]. In two studies, at the beginning, the study group consisted of subjects with a positive result of specific celiac antibodies;—i.e., antibodies to tissue transglutaminase (TG2) in immunoglobulin class A (IgA) and/or TG2 IgG—and CD was diagnosed in these patients during the study [17,18]. The groups of patients in the analyzed studies were also not homogeneous. In four out of the six analyzed original papers, the study groups included both adults and children [15,16,17,18]. However, in one of them, there were children over 14 years of age and adults [17]; the second included subjects from 1 to 20 years of age [16]. In the other two studies, only children [12] and adults [14] were analyzed.

The number of studies included in the current systematic review appeared to be too small to draw generalized conclusions about the prevalence of A-IgE in patients with CD. Additionally, none of the studies confirmed an allergy to a specific allergen with the use of a double-blind, placebo-controlled challenge. Therefore, based on the found and analyzed papers, we could only draw conclusions about the frequency of sensitization in people with CD.

In the subjects with CD included in this analysis, sensitization (based on the measurement of the concentration of specific immunoglobulin E (sIgE) in the blood serum) ranged from 16.6–20.0% when sIgE was tested to more than one allergen [12,14]. The most frequently tested allergens were those containing gluten (e.g., wheat, rye, and barley) [15,16,18]. The analysis showed that the frequency of a wheat allergy in subjects with CD ranged from 4.0–7.0% [15,16]. The incidence of sensitization to rye and barley in CD subjects was 10.8% and 5.4%, respectively [16]. The case studies presented in Table 3 also reported that the most common allergen to provoke allergy symptoms in CD patients was wheat. Typically, an IgE-mediated reaction to this allergen appeared in early childhood and was overcome within 3–5 years of age, but in 3 out of 15 reported cases, a wheat allergy developed at a later age [19,20,21]. 

Other food allergens against which sensitization occurred in CD patients included cow’s milk, hen’s eggs, lupines, buck flour, barley, oats, lentils, fish, peanuts, rye, and corn [20,22,23,24,25,26,27]. Although a wheat allergy/sensitization was the most common in CD patients, especially in children, sIgE to other allergens was also found in sensitized CD subjects [14]. Ciacci et al. assessed the incidence of A-IgE in adult patients with CD (n = 1044), their relatives (n = 2752), and spouses (n = 318; as a control group) [14]. The study was based on questionnaires distributed among patients at the moment of a CD diagnosis. Subjects reporting any allergy underwent skin-prick tests and the determination of total IgE and sIgE in serum against 20 allergens, including inhaled (*Graminacee*, *Parietaria officinalis*, *Dermatophagoides*, and *Alternaria*) and food (milk, eggs, fish, shellfish, nuts/peanuts, tomatoes, citrus fruits, and soya) allergens. Among the study subjects, 173 (16.6%) with CD, 523 (19%) relatives, and 43 (13.5%) spouses had an allergy to at least 1 allergen, most often to pollen (53; 30.64%), mold (49; 28.32%), vegetables (12; 6.94%), cosmetics (11; 6.36%), and fish (6; 3.47%). Cudowska et al. [12] studied a group of 59 children aged from 10 months to 17 years (mean age 8.1 +/− 4.4 years) with diagnosed CD and demonstrated the presence of sIgE in more than 20% (n = 12) of the children. The children were most frequently sensitized to airborne allergens (66.7%), then to food allergens (58.3%) (a few subjects were sensitized both to airborne and food allergens). Of the airborne allergens, the most frequently sensitizing were house dust mites (6/12; 50%), grass (5/12; 41.7%), and birch pollen (4/12; 33.3%). Of the food allergens, the most frequently sensitizing were peanuts (5/12; 41.7%), cow’s milk protein (3/12; 25%), and hen’s egg protein (2/12; 16.7%). 

It needs to be highlighted that in the analyzed studies, various tests were used to diagnose sensitization/allergies, from self-reporting questionnaires of allergy symptoms through skin-prick tests and the determination of sIgE in blood serum to the use of the most modern tool, which was molecular allergy diagnostics. Among the allergen molecules, serum sIgE was determined to Tri a 14, Tri a 19, and Tri a aA_TI (the inhibitor a1 α-amylase) from wheat, which may be associated with an anaphylaxis risk. Phl p 1, Phl p 5, Phl p 7, and Phl p 12 from timothy grass were associated with an inhalation allergy to this grass [15,18]. 

Among the analyzed original studies, two of them did not confirm the possibility of A-IgE in CD [17,18]. Enroth et al. did not find subjects with CD and A-IgE in their survey conducted as part of a health-state assessment in 1068 subjects above 14 years of age [17].

Similarly, Spoerl et al. [18], on the basis of a retrospective analysis of laboratory test results performed from 2010–2016, indicated that a wheat allergy did not seem to be related to CD. Nevertheless, of note is a comparison of the number of serological tests for CD with the number of sIgE determinations for wheat. Only 1% of patients tested for CD underwent an sIgE determination for wheat, despite the fact that approximately 20% of the patients tested for CD had intestinal symptoms (bloating or cramps) indicative of a wheat allergy. These findings were emphasized by the authors, who implied the underestimation of the allergy incidence in subjects with CD [18].

### 3.2. Clinical Manifestation of A-IgE in CD Patients 

The analysis of the studies showed that A-IgE in patients with CD manifested similarly to non-CD subjects. The case studies (Table 3) reported that CD subjects presented with allergic symptoms such as diarrhea, abdominal pain, vomiting, urticaria, hypotension, a loss of consciousness, facial angioedema, dyspnea, coughs, or anaphylaxis. The cases also included seasonal rhinitis, asthma, and atopic dermatitis [20,24,27,28,29,30]. 

The symptoms of A-IgE in CD patients were also analyzed in selected original papers [12,14,16]. Lanzarin et al., in their group of 74 children and teenagers with CD, confirmed the occurrence of asthma, allergic rhinitis, and atopic dermatitis in more than 17%, 13.5%, and 5.4% of the study subjects, respectively [16]. Ciacci et al. presented that atopic dermatitis occurred three times more often in subjects with CD (3.8%) and two times more often in their relatives (2.3%) than in their spouses (1.3%) [14]. Furthermore, no change in the incidence of allergies in the study subjects with CD was demonstrated after a year of adherence to a GFD [14]. Cudowska et al. [12] also presented that A-IgE in CD children often manifested as atopic dermatitis. It is noteworthy, however, that in this study, 1/3 of CD children had atopic dermatitis, but the symptoms were only caused by food allergens in 5 of them. On the other hand, the authors noticed that other skin diseases such as urticaria or herpetiform dermatitis could be claimed to be a skin form of CD, and gastrointestinal symptoms could be misinterpreted as those only related to CD. 

Another group of allergic symptoms in patients with CD are gastrointestinal symptoms. Cudowska et al. [12] showed that almost 42% of CD children reported gastrointestinal symptoms despite using a GFD. The similarity of symptoms in A-IgE and CD can make it difficult to determine which of the diseases developed first, as shown in the descriptions from a few cases. Borghini et al. described a case of a 25-year-old woman who was diagnosed with both diseases at the same time [29]. Similarly, in the oldest reported case (1986) [31] of A-IgE in a 3-month-old infant, it was difficult to decide which disease developed first. Symptoms such as diarrhea and weight loss occurred after the introduction of a cow’s milk and wheat diet [31]. Among the case studies presented in Table 3, there were 12 studies describing a total of 15 cases of A-IgE that developed after a diagnosis of CD. However, in 4 of these subjects, an allergy was also present before the CD diagnosis; after the diagnosis, an allergy to new allergens developed [20,27,30].

Regarding the fact that only 4 out of 6 original studies were designed in a way that enabled the reporting of effect sizes, with a low precision of the effect sizes and a high variability of the data, a calculation of the bias with the use of statistical methods was not possible. An overall risk of publication bias was plausible, but unclear. 

**Table 3:** Comparison of case reports of IgE-mediated allergies in subjects with CD included in the literature review.

No.	Autor/Publication Year/n ^1^	Age of CD Diagnosis/Gender	Symptoms ^2^	Confirmed Allergens	Other Concomitant Allergic Diseases
1	Kuitunen et al. [31]; 1986; n = 1	10.9 years/boy	Vomiting; diarrhea	Cow’s milk	
2	Rotiroti et al. [22]; 2007; n = 1	23 years/woman	Generalized urticaria, upper airway angioedema, wheezing, laryngeal edema, vomiting, profound hypotension, and loss of consciousness	Lupines	
3	Torres et al. [23]; 2008; n = 1	4 years/girl	Abdominal pain, gastric fullness, flatulence, and vomiting immediately	Wheat, gliadin, barley, and oat	
4	Sánchez-García et al. [24]; 2011; n = 1	2 years/girl	Abdominal pain, facial urticaria, and generalized urticaria	Cow’s milk; eggs	AD; asthma
5	Wong et al. [19]; 2014; n = 1	18 months/girl	Urticaria, cough, shortness of breath with accidental exposures to wheat, tingly mouth, and wheezing	Wheat	
6	Heffler et al. [25]; 2014; n = 1	37 years/woman	Chronic urticaria	Buckwheat flour	
7	Dondi et al. [28]; 2015; n = 1	9 years/boy	Oral allergy syndrome, respiratory impairment, hives, angioedema, abdominal pain or vomiting, and mild-to-moderate anaphylactic reactions	Cow’s milk	AD, inhalant allergies, and asthma
8	Martín-Muñoz et al. [20]; 2016; n = 2	25 months/boy	Wheezing, urticarial, lip edema, vomiting, and bronchospasm	bdCD: hen’s eggs, lentils, and fish adCD: wheat flour, hake, eggs, and lentils	AD, spring rhinitis, and asthma
		14 months/girl	Nasal pruritus, facial angioedema, dyspnea, and cough	Wheat flour	Spring rhinitis
9	Micozzi et al. [30]; 2018; n = 2	12 months/nd	Delayed growth, abdominal pain, vomiting, sneezing, and lacrimation	bdCD: hen’s eggs, cow’s milk adCD: wheat flour	Rhinoconjunctivitis; asthma
		6 months/nd	Delayed growth; eyelid angioedema	bdCD: hen’s eggs, cow’s milk adCD: wheat flour	Rhinoconjunctivitis; asthma
10	Borghini et al. [29]; 2018; n = 1	25 years/woman	Swelling, abdominal pain, diarrhea, and weight loss	Wheat	AD; erythematous skin lesions
11	Mennini et al. [27]; 2019; n = 1	3 years/boy	Anaphylaxis	bdCD: hen’s eggs adCD: wheat	AD, rhinitis, and asthma
12	Lombardi et al. [26]; 2019; n = 2	5 years/girl	Generalized urticaria, lip swelling, abdominal, pain, diarrhea, vomiting, and respiratory distress	Wheat	
		26 years/woman	Hypotension, generalized urticaria, lip swelling, abdominal pain, diarrhea, vomiting, and dyspnea	Wheat	

^1^ n: Number of patients described. AD: atopic dermatitis; bdCD: before CD diagnosis; adCD: after CD diagnosis. ^2^ Symptoms associated with an allergic reaction to the allergen to which the allergy occurred after the diagnosis of CD.

## 4. Discussion

The analysis conducted on the basis of the literature indicated that a possible coexistence of A-IgE in CD subjects should be considered. Allergies in CD subjects manifested similarly to non-CD subjects; e.g., with atopic dermatitis, vomiting, urticaria, angioedema, or anaphylactic shock. The literature most often describes subjects with CD as allergic to wheat, which may have been because this allergen was the most frequently tested in the studies. Screening for allergies in subjects with CD should be considered, especially in those cases where symptoms persist after introducing a GFD. There are no available original studies on the effect of a GFD in subjects with CD on the development of a wheat allergy or an allergy to other gluten-containing cereals. A few reported cases indicated that the elimination of gluten-containing foods may induce the appearance of sIgE against these foods [26,27,30]. A hypothesis was even developed that the interrupted and accidental consumption of gluten by patients with CD may promote sensitization [30]. An example that could confirm this hypothesis was the case of a girl who was diagnosed with CD at the age of 6. After many years of a strict GFD, at the age of 15, the girl started to consume gluten occasionally, which could have contributed to the development of anaphylaxis when she ate pita bread. In the case report, the authors hypothesized that occasional gluten consumption by subjects on a GFD was likely to contribute to the development of an allergy to wheat [26]. Elimination diets, including a GFD, may lead to a reduced tolerance to the products excluded from the diet. This hypothesis was also supported by the described case of a 13-year-old boy with CD and a GFD who developed anaphylaxis after accidentally ingesting gluten [27]. Another hypothesis that could be considered after analyzing the literature regarding the occurrence of A-IgE in subjects with CD was that it may be that patients with CD are already sensitized to wheat. Embarking on a GFD may lead to a loss of tolerance to wheat and, thereby, to the development of a wheat allergy [30]. Further investigations into this issue are necessary because avoiding eating wheat for subjects with CD and a wheat allergy may lead to fatal anaphylaxis [28]. Importantly, food products introduced as substitutes for gluten-containing cereals may also be a source of allergens responsible for the development of anaphylaxis in sensitized patients with CD. A case of a patient with CD who developed anaphylaxis after eating gluten-free pasta was described. It was established that sensitization to lupines caused the symptoms [22]. In the cases of unexplained anaphylaxis, it is essential to establish if the patients with CD were not allergic to food products introduced as substitutes for gluten-containing cereals and to monitor the risk of sensitization in these patients with CD.

The diagnosis of A-IgE in patients with CD may be difficult due to allergic symptoms being masked by symptoms interpreted as those related to CD. There are two main types of clinical A-IgE manifestations occurring in CD: skin and gastrointestinal symptoms. Unfortunately, both groups of symptoms are also typical in the course of CD without an allergy [3]. Therefore, it is important to consider allergies in patients with CD who fail to clinically improve with the introduction of a restrictive GFD.

The diagnostic tests used may also lead to a misinterpretation of the results and a failure to diagnose allergies. In the case of a 15-year-old girl with anaphylaxis after the consumption of pita bread, skin-prick tests gave a positive result for gliadin (8 mm), but a negative result for wheat extract [26]. It must be emphasized that the diagnostic tests are based on various allergen extracts that are soluble in water and that not all allergens prepared in this way contain allergic proteins. Several allergic proteins only dissolve in the presence of detergents or alcohol [32,33]. As wheat proteins are poorly water-soluble, the diagnostics based on their extracts may fail to establish the allergic source responsible for the symptoms. A new tool enabling a more detailed analysis of sIgE to particular allergen proteins and highly facilitating the diagnosis of wheat allergies in patients with CD is precision allergy molecular diagnosis (PAMD@) [34]. After the discovery of sIgE, the use of tests detecting antibodies to allergen molecules has become another milestone in the diagnosis of allergies. PAMD@ provides more information than a diagnosis based on extracts and it enables both a differentiation between primary sensitizations and sensitizations resulting from cross-reactions and the determination of the risk of anaphylaxis [35]. Therefore, often only this tool can precisely establish what the cause of an allergic response is [32,34]. In the reported cases, apart from the determination of serum sIgE to the whole wheat extract, more and more often its particular proteins such as Tri a 14 (nsLTP), Tri a aA_TI (α-amylase), and Tri a 19 (ω-5-gliadin) had been determined [20,23,26,27,28,30]. In the case of an allergy to wheat in a girl with CD diagnosed when she was 5, which was reported by Lombardini et al., particular wheat molecules were also assessed. It was confirmed that she had serum sIgE to gliadin and ω-5-gliadin [26]. ω-5-gliadin is a marker of both a primary allergy to wheat and of severe reactions in children with an allergy to wheat, as well as a marker of wheat-dependent exercise-induced anaphylaxis [32]. PAMD@, as a new diagnostic method, may help diagnose patients with CD who, despite the use of a restrictive GFD, still experience bothersome symptoms resulting from their food intake.

## 5. Conclusions

Our study was the first systematic review on allergy occurrence in CD patients. Although there are few studies assessing the occurrence of A-IgE in subjects with CD, the current systemic review indicated the possible coexistence of A-IgE in CD. However, the occurrence of A-IgE in subjects with CD is a not fully understood problem; this requires further studies in particular populations as well as studies that would explain a possible pathomechanism of a coexistence of these two diseases.

## Figures and Tables

**Table 1:** The number of articles found as a result of searching article titles in the PubMed database using the keywords “allergy&celiac/coeliac”, “sensitization&celiac/coeliac”, and “anaphylaxis&celiac/coeliac” in the form of a conjunction as of 28 December 2022.

Keywords	All Articles in PubMed Included in the Analysis	Original Studies	Systematic Reviews	Case Studies	Review Articles	Inconsistent with the Search Topic	Other ^3^
“allergy&celiac/coeliac”	48 ^1^	4	0	9	5	17	13
“sensitization&celiac/coeliac”	6 (+1 ^2^)	2	0	0	0	1	3
“anaphylaxis&celiac/coeliac”	3	0	0	3	0	0	0
	57 (+1 ^2^)	6	0	12	5	18	16

^1^ A total of 53 articles containing the words “allergy&celiac/coeliac” were found, but 4 of them, although containing the searched words, did not refer to the research topic after reading the content and one was dated 2023. ^2^ One article showed up both with the use of “sensitization&celiac” and with “allergy&celiac” because its title contained all three words “allergy&sensitization&celiac”. ^3^ Others: chapter of a book, abstract of a conference, symposium report, letter to editor, unavailable articles (e.g., absent in the search engine or an article in a language other than English).

**Table 2:** Summary of original studies assessing the coexistence of allergy/sensitization and CD.

No.	Study	Number of All Patients Tested/Number with CD	Age Cohort (Mean Age, y)	Region/Type of Study/Period	Tests Used for Diagnosis of Allergy/Sensitization	Tested Allergens	The Prevalence of Allergies/Sensitivities in Celiac Disease
1	Ciacci et al. [14]; 2004.	4114/1044 CD	Adults	Italy; prospective; 1992–2002	Reporting allergy symptoms; sIgE/nd; SPT/Stallergenes Srl, Saronno, Italy	*Graminacee, Parietaria officinalis, Dermatophagoides*, and *Alternaria*; skin: nickel, chrome, latex, cosmetics, soaps, and dye; milk, eggs, fish, shellfish, nuts/peanuts, tomato, citrus fruits, and soya; miscellaneous: all the reactions provoked by less common antigens (for example, pigeon’s feathers, cloves, cinnamon, and olive tree pollen)	16.6% had sIgE for minimum of one tested allergen
2	Armentia et al. [15]; 2008.	88/57 CD	Children; adults	Spain; prospective; ND ^1^	SPT/ALK-ABELLO Laboratories, Madrid, Spain	Pollen, mites, molds, and different foods;	7.0% sIgE to wheat, but no patient in this group had IgE to other food allergens
					sIgE/Pharmacia CAP System FEIA, (Uppsala, Sweden)	wheat, barley, and rye flours and a battery of food allergens: whole milk, α-lactalbumin, β-lactoglobulin, casein, eggs (white and yolk), legumes, nuts, and fish; Tri a 14, Tri a aA_TI (inhibitor a1 α-amylase (included CM3))	
3	Enroth et al. [17]; 2013.	1068/24 people with TG2 IgA and/or TG2 IgG	Children (14+); adults	Sweden; prospective; 2006 and 2009	Self-reported allergy	Grass/pollen, cow’s milk, gluten, fur, fish, dust, cold air, mold, organic solvents, medicines, and others	No people with allergies were found in the study group with CD
					sIgE/ImmunoCAP^®^, F×5, and Phadiatop Thermo Fisher Scientific/Phadia, Uppsala, Sweden	ND	
4	Spoerl [18]; 2019.	2965/128 subjects with positive tTG IgA	Children; adults	Switzerland; retrospective; 2010–2016	sIgE/ImmunoCAP^®^ Thermo Fisher Scientific/Phadia, Uppsala, Sweden)	Wheat extract, molecule Tri a 19, and molecular timothy grass Phl p 1, Phl p 5, Phl p 7, and Phl p 12	Wheat allergy did not seem to be associated with CD
5	Lanzarin et al. [16]; 2020.	74/74 CD	1–20 years of age	Brazil; prospective; NR	sIgE/ImmunoCAP^®^, Thermo Fisher Scientific/Phadia, Uppsala, Sweden))	Wheat, rye, barley, and malt	Frequency of sensitization to wheat, rye, barley, and malt among CD patients was 4, 10.8, 5.4, and 2.7%, respectively
6	Cudowska et al. [12]; 2021.	59/59 CD	Children (average age 8.1)	Poland; retrospective; 2016–2018	sIgE/Polycheck; Biocheck GmbH, Münster, Germany	20 major food and airborne allergens	20.3% children were sensitized
					SPT/Allergopharma and Nexter	Milk, eggs, soy, wheat, pork, cod, citrus fruits, peanuts, and airborne allergens	

^1^ ND: no data.

## Data Availability

The data presented in this study are available on request from the corresponding author.
